# A shorter path to circumventing the low reproducibility of SERS spectra through variable screening and optimization methodologies

**DOI:** 10.1007/s00604-026-08306-x

**Published:** 2026-07-28

**Authors:** Antonio Morais Neto, Maycom Cezar Valeriano, Paula Homem-de-Mello, Bruno Guzzo da Silva, Mónica Benicia Mamián-López

**Affiliations:** Universidade Federal do ABC, Av. dos Estados, 5001, Santo André - São Paulo, 09210-580 Brazil

**Keywords:** Design of experiments, Quantitative SERS, Vibrational spectroscopy, Variable screening

## Abstract

Currently, several SERS methodologies are focused on quantitative goals, and, as they move toward becoming a suitable analytical alternative, their main drawback remains the low reproducibility of SERS signals. As a result, strategies to circumvent this are highly desirable, but given the number of experimental variables that affect reproducibility, the task can sometimes be endless. Here, we selected a series of C5-substituted uracil derivatives (5-fluoro-, 5-chloro-, and 5-bromouracil) adsorbed on silver nanoparticles. These compounds are of analytical and pharmacological interest due to their role as biomarkers, pharmaceuticals, or environmental pollutants. They are expected to be found at very low levels in bioanalytical matrices; thus, to reach low limits of detection, they were paired with adenine, which has a well-known high affinity for silver nanoparticles. Considering the complexity of these pairs and the experimental variables that affect SERS reproducibility, a two-stage strategy was adopted to select the best experimental conditions and minimize the standard deviation (STD) of the signals from the three base pairs. This included a variable screening procedure followed by an optimization stage. After optimization, the STD values decreased by 100-, 300-, and 1000-fold for 5-fluorouracil, 5-bromo, and 5-chlorouracil, respectively. The validation experiments yielded STD values at the same order of magnitude as those achieved under the optimized conditions, indicating that our approach can be applied to improve signal reproducibility. In perspective, this strategy can be easily adapted to other SERS-based systems, especially for analytical purposes.

**Graphical abstract:**

**Figure:**
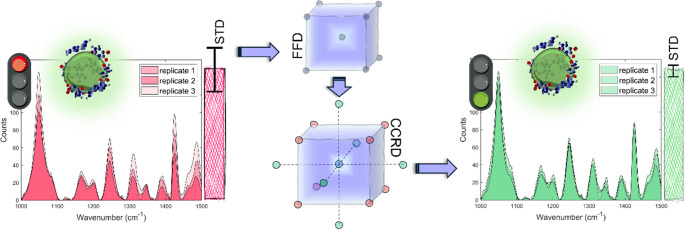

**Supplementary Information:**

The online version contains supplementary material available at 10.1007/s00604-026-08306-x.

## Introduction

One of the most promising alternatives to conventional Raman Spectroscopy is Surface-Enhanced Raman Spectroscopy (SERS), based on the so-called effect, which is widely used nowadays for quantitative purposes. Briefly, the SERS effect allows the enhancement of the Raman scattering when a target molecule is in the proximity or in the hotspot regions of noble metal nanostructures [1–3]. SERS, as a technique, is almost independent of Raman, despite both measuring inelastic light scattering by molecules. Currently, several SERS methodologies are being developed, and many focus on quantitative goals. On the path to becoming a suitable analytical alternative, its main drawback has been the low reproducibility of SERS spectra, especially when metal nanoparticles are used as an enhancer surface. Also, considering the number of experimental variables that affect the signal reproducibility, the task can sometimes be endless [4–6]. 

Despite significant advances in achieving substantial Raman signal enhancement, the reproducibility of SERS spectra remains a bottleneck in analytical applications. This is strongly linked to the synthesis of colloidal nanostructures [7], since their size distribution is highly influenced by small changes in experimental conditions, such as temperature, stirring speed, and the reducing agent, among others. Diverse strategies have been applied to improve reproducibility, with many focusing on more controlled synthesis methods to achieve nanostructures with narrow size dispersion [8, 9]. Other strategies involve functionalizing nanoparticles or embedding them in stabilizing media, such as polymers or gels [10, 11]. Machine learning tools have also been a major ally in the path to developing mainly quantitative SERS methodologies, with numerous works showing their potential [12, 13]. Nevertheless, reaching near-optimal conditions is highly time- and reagent-consuming and can cause significant delays in achieving quantitative objectives.

An effective approach that can drastically reduce the path length to more reproducible SERS spectra is the Design of Experiments (DOE) methodology, which has recently been applied in a few SERS applications [14, 15]. Some studies have reported applying this tool to optimize SERS experiments, primarily to improve colloid synthesis [15, 16] or to quantify a specific species [14]. This tool can significantly reduce time and reagent consumption, enabling visualization of the complete landscape of the variable effects [17, 18].

The 5-fluoro, 5-chloro, and 5-bromouracil (5-FU, 5-ClU, and 5-BrU), studied here, are of analytical and pharmacological interest due to their role as biomarkers, pharmaceuticals, or environmental pollutants. They are expected to be present at very low levels in bioanalytical matrices. However, when measured alone, the SERS response of their main backbone, uracil, is usually weaker than that of other nucleotides, as discussed in the literature [19, 20], and this is probably associated with its orientation on the metal surface [21]. One way that could induce a more favorable orientation is to pair it with an analog molecule, such as adenine, which has a well-known high affinity for silver nanoparticles. It has been previously suggested in the literature that pairing adenine to a pyrimidine could modify its SERS signal, especially the ring stretching modes, as we also show in this work [22, 23]. Considering the complexity of these pairs and the experimental variables affecting SERS reproducibility, a shortcut to an optimized set of experimental conditions to minimize the STD of the signals across the three base pairs was adopted. In the first stage, a variable-screening procedure selected two of the four variables; in the optimization stage, the best conditions were identified. Finally, validation experiments under the optimized conditions were conducted, and the validity of the chosen vibrational mode was verified through exploratory PCA analysis.

## Experimental

### Material and equipment

5-fluorouracil (≥ 99% Sigma-Aldrich), 5-chlorouracil (≥ 99%, Sigma-Aldrich), 5-bromouracil (≥ 98%, Sigma-Aldrich), silver nitrate (≥ 99%, MERCK), sodium citrate dihydrate (≥ 99%, Sigma-Aldrich), sodium chloride (≥ 99,95%, Fisher Scientific), and adenine (≥ 99% Sigma-Aldrich) were used. A Triple Raman Spectrometer T64000 (Horiba Jobin-Yvon) operating with a 532 nm laser and 10x objective lens, and a Renishaw inVia confocal microscope equipped with an excitation line at λ_0_ = 532 nm and a 20x long-working distance objective were used. The base pair solutions were obtained by mixing their separate solutions to form adenine-5-fluorouracil (Ade-5FU), adenine-5-chlorouracil (Ade-5ClU), and adenine-5-bromouracil (Ade-5BrU). Milli-Q water was used to prepare all samples.

### Synthesis of silver nanoparticles (AgNPs)

The AgNPs were prepared by the Lee and Meisel method [24], which follows the reduction of silver with sodium citrate (1%). Briefly, 4.6 mL of 11 mM silver nitrate (AgNO_3_) was diluted in 100 mL of ultra-pure water. The solution was stirred continuously and heated until the water was nearly boiling. After this, 3.0 mL of 1% sodium citrate was slightly added. The solution was stirred continuously until it turned yellowish-green.

### Characterization of AgNPs

The AgNPs were characterized by UV-Vis spectroscopy (Shimadzu UV-3600) to measure their extinction spectrum and by Transmission Electron Microscopy (TEM) to estimate their morphology and size (Talos F200X, G2). Dynamic Light Scattering (DLS) analysis, polydispersity index, and zeta potential were carried out using a Zetasizer Nano ZS equipment (Malvern Instruments Co). The measurements were performed in aqueous medium at 24.9 °C, using a disposable capillary zeta cell.

### Variable screening and optimization

Factorial designs involve combinations of levels across two or more variables. While a full factorial design requires the number of experiments to be 2ᵏ (where k is the number of experimental variables being evaluated) [25–29], in a fractional factorial design, the number of experiments is given by 2^k−n^, where n is the fraction value, and k is the number of experimental variables [25, 29]. This type of design is useful when studying a large number of experimental variables (k ≥ 4), as it allows the selection of the more significant variables, which can then be used in a second, optimization stage. For this purpose, there are various possibilities of DOE methodologies, with the Central Composite Rotatable Design being a suitable and common option [30]. This methodology is built with a number of experiments equal to 2^k^ + 2k + 3, where 3 corresponds to the replicates at the central point, and 2k are the axial points whose distance from the central point is defined by an α parameter.

#### Fractional Factorial Design

The most appropriate methodology for selecting the important variables (when their number is 4 or more) is the Fractional Factorial Design (FFD) [27]. This choice is particularly suitable during the development of SERS methodologies, as multiple experimental variables influence signal reproducibility [31]. Here, the FFD applied was 2^(4−1)^ + 3 = 11 experiments. The samples were prepared according to the values outlined in Table 1. The AgNPs were activated with NaCl beforehand, and then, 100 µL of the previously formed base pairs was added to 1000 µL of colloid. SERS spectra were measured in triplicate, and the experiments were conducted in random order. The experimental results were analyzed using Protimiza Experimental Design^®^ software, with a 10% significance level.

The four variables at this stage were the volumes of adenine (5 × 10^− 5^ mol L^− 1^), 5-XU (1 × 10^− 4^ mol L^− 1^), NaCl (5 × 10^− 2^ mol L^− 1)^, and colloidal AgNPs. Nevertheless, although factors such as incubation time (AgNPs with analyte or aggregating agent), ionic strength, or pH can also be influential in this kind of system, these conditions were not included in the DOE methodologies due to the reasons below:

- Incubation time is not a critical parameter in our case, since the colloidal AgNPs are quite stable during a typical analysis time (45 min/sample at longest). This stability was evaluated using the Surface Plasmon Resonance (SPR) band shown in Figure S2c, where the spectrum of AgNPs activated with NaCl and mixed with the analyte remains stable for at least 45 min, which is a sufficiently long interval to measure a sample.
- Ionic strength: In our system, the ionic strength depends on the concentration of the aggregating agent (NaCl). The concentration used here was also based on the SPR band behavior, as a significant increase under these conditions is expected to cause aggregation of the AgNPs, which is easily detected by a bathochromic shift of the SPR maximum (See Figure S2b). In practice, strong aggregation of the colloid damps the SERS effect; no spectrum will be obtained [32]. As for our system, a low NaCl concentration causes a slight, controllable aggregation, enough to intensify the Raman signal; this was not a variable of interest.
- pH: This parameter was kept at the value given by the AgNPs colloid (pH = 3.7), as at this condition, the adenine is mostly non-protonated, which favors its orientation, hence its SERS spectrum. An additional reason to maintain the medium’s natural pH was that adding buffers would alter the ionic strength, which would in turn affect the colloid’s stability, as discussed earlier.

**Table 1:** Independent variables for the FFD experiments and STD values for the bands at 789, 775, and 798 cm^− 1^

	Independent variables (Volume)*				STD of the intensity at the pyrimidine mode of each pair		
Exp.**	Adenine (µL)	5-XU (µL)	NaCl (µL)	AgNPs (µL)	Ade-5FU (789 cm^− 1^)	Ade-5ClU (775 cm^− 1^)	Ade-5BrU (798 cm^− 1^)
1	50 (-1)	50 (-1)	20 (-1 )	1000 (-1)	6.40	**1.75**	71.66
2	150 (+ 1)	50 (-1)	20 (-1 )	2000 (+ 1)	2.49	12.45	16.02
3	50 (-1)	150 (+ 1)	20 (-1 )	2000 (+ 1)	10.40	2.91	41.62
4	150 (+ 1)	150 (+ 1)	20 (-1 )	1000 (-1)	5.72	7.20	17.57
5	50 (-1)	50 (-1)	100 (+ 1)	2000 (+ 1)	2.05	3.39	**2.67**
6	150 (+ 1)	50 (-1)	100 (+ 1)	1000 (-1)	**1.87**	91.49	22.40
7	50 (-1)	150 (+ 1)	100 (+ 1)	1000 (-1)	4.04	8.70	14.04
8	150 (+ 1)	150 (+ 1)	100 (+ 1)	2000 (+ 1)	2.05	2.15	13.12
9	100 (0)	100 (0)	60 (0)	1500 (0)	2.57	16.32	18.05
10	100 (0)	100 (0)	60 (0)	1500 (0)	3.67	12.75	8.31
11	100 (0)	100 (0)	60 (0)	1500 (0)	2.93	13.83	26.08

*Coded values in parentheses. ** *Exp. *experiment

#### Central Composite Rotatable Design (CCRD)

Two input variables were selected for the CCRD optimization (Table 2): volumes of 5-XU (X = F, Cl, and Br) and NaCl, and a volume range was established for each base pair. For the Ade-5FU and Ade-5ClU base pairs, the variable values are detailed in Table 3. For the Ade-5BrU, the variable values are given in Table 4.

**Table 2:** CCRD variable levels for each experiment and the corresponding STD values for the bands at 789, 775, and 798 cm-1 for Ade-5FU, Ade-5ClU, and Ade-5BrU, respectively

	Volume (coded values)		STD of the intensity at the pyrimidine mode of each pair (counts)		
Exp.*	5-XU (µL)	NaCl (µL)	Ade-5FU	Ade-5ClU	Ade-5BrU
1	-1	–1	2.17	**0.08**	0.79
2	+ 1	–1	0.15	0.26	0.69
3	-1	+ 1	0.61	0.68	0.34
4	+ 1	+ 1	0.50	3.02	**0.29**
5	-1.41	0	**0.10**	1.69	0.51
6	+ 1.41	0	0.72	1.66	0.52
7	0	-1.41	0.77	1.56	0.69
8	0	+ 1.41	0.27	0.33	0.60
9	0	0	0.62	6.36	0.59
10	0	0	1.01	4.05	0.54
11	0	0	0.28	0.18	0.93

*Exp. experiment

**Table 3:** Experimental and coded variable values for the base pairs Ade-5FU and Ade-5ClU

Variables	Experimental and coded values				
	-1.41	-1	0	1	1.41
5-FU and 5-ClU(µL)	3.43	20	60	100	116.6
NaCl (µL)	3.43	20	60	100	116.6

**Table 4:** Experimental and coded values for the base pair Ade-5BrU

Variables	Experimental and coded values				
	-1.41	-1	0	1	1.41
5-BrU	79.3	100	150	200	220.7
NaCl (µL)	3.43	20	60	100	116.6

The choice of the ranges above depended on the variable screening procedure and was made to minimize the STD values. It is important to highlight that for the Ade-5BrU pair, the range was narrower. This was due to higher values of this variable, which caused a destabilization of the AgNPs. This is probably related to the higher affinity of 5-BrU towards AgNPs, and its characteristic as a more effective charge donor, compared to 5-ClU and 5-FU. A deeper explanation of this is not included in this study, but can be found in our previous work [33].

The STD calculation followed Eqs. 1,

1 $$\:STD=\sqrt{\frac{{\left({X}_{i}-\underset{\_}{X}\right)}^{2}}{n-1}}$$

where *X*_*i*_ is the absolute SERS intensity value for a given band, $\:\stackrel{-}{X}$ is the mean intensity across the three replicates at the same wavenumber, and *n* is the number of measurements. The choice of the pyrimidine ring mode [34] was due to its representativeness in SERS spectra of base pairs and their derivatives, and because it is distinctive of both adenine and 5-XU species. The experimental results were analyzed using Protimiza Experimental Design^®^ software, with a significance level of 5%. Analysis of Variance (ANOVA) was used to assess the fit of a quadratic polynomial model.

### Sample conditioning and spectra acquisition

The samples were prepared according to the values in Tables 1, 2, 3 and 4 and in the following order: (a) Formation of base pairs by mixing adenine and 5-XU solutions; (b) activation of AgNPs with NaCl(aq); (c) mixture of the activated AgNPs with the previously formed base pairs in a 10 to1 volume ratio. Control experiments were run by measuring the SERS spectra of each 5-XU, adenine, and the Ade-5XU pairs separately.

The acquisition of the SERS spectra was carried out using a Raman Spectrometer T64000 by Horiba Jobin-Yvon (Excitation wavelength: λ_0_ = 532 nm, laser power at the sample: 8 µW, 10x long-working-distance objective lens, with 10 accumulations of 6 s each). Also, a Renishaw InVia confocal Raman Microscope was used to acquire part of the spectra (Excitation wavelength, λ_0_ = 532 nm, nominal laser power 500 mW, 20x long-working-distance objective lens, with 5 accumulations of 5 s each). For both instruments, the Raman signal was calibrated using a Si wafer, whose maximum was centered at 520 cm^− 1^, and the measured spectral region ranged from 200 to 1800 cm^−^¹. To avoid significant differences in STD calculations from the bands’ intensities, the response was absolute and calculated from the nominal Raman intensity value. Both the optimization and validation stages were conducted using only the Spectrometer T64000 by Horiba Jobin-Yvon.

### Data processing and multivariate analysis

All spectra were processed in Matlab environment (Version 2023a). The datasets were pre-processed with baseline correction (Automatic Whittaker Filter, asymmetry = 1e^− 06^ and λ = 100), a Savitzky-Golay smoothing filter (order = 0, window = 5 pt), and mean centering. Multivariate exploratory analyses were conducted with PLS Toolbox Version 9.1 (2022).

#### Principal component analysis

The Principal Component Analysis (PCA) method reduces the dimensionality of the data matrix by projecting the data onto a new coordinate system called principal components (PCs). This enables visualization and interpretation of trends or patterns in two or three dimensions at a time [35, 36]. In PCA, the raw, preprocessed X_n, m_ matrix is decomposed into a two-matrix product, T_n, k_ and L_k, m,_, as shown in Eqs. 2,

2 $$\:X={t}_{1}{l}_{1}+{t}_{2}{l}_{2}^{T}+{{t}_{3}l}_{3}^{T}+\dots\:+{t}_{n}{l}_{k}+E=T{L}^{T}+E$$

where t_k_ and l_k_ are the scores and loadings of each PC, respectively, and the k index represents the matrix rank or the number of PCs in the new, reduced dimension. The scores are the coordinates in the new system of axes (or PCs), while the loadings show the contribution of each variable in the original matrix to each PC. Finally, the E matrix contains residual, not modeled information [35].

## Results and discussion

### Characterization of AgNPs

**Fig. 1:**
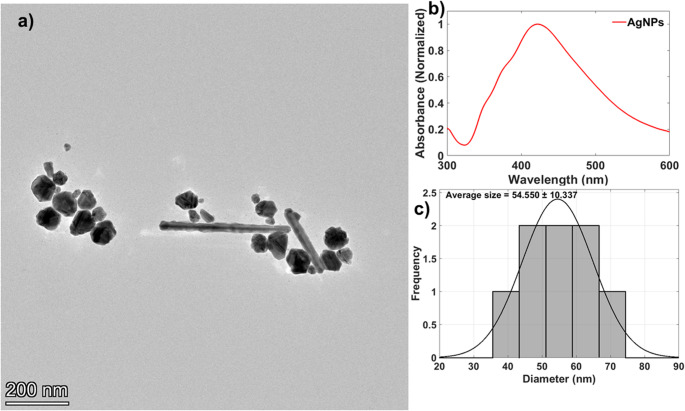
(**a**) TEM images of AgNPs, (**b**) Uv-Vis spectrum of AgNPs, and (**c**) AgNPs size histogram

The characterization of the AgNPs by TEM revealed a semispherical morphology. Their UV-Vis extinction spectrum showed a single SPR band at 420 nm, consistent with reports for classical AgNPs in the literature [37]. The average size of the AgNPs was estimated to be approximately 55 nm. Both images and the extinction spectrum are shown in Fig. 1. Additionally, batch-to-batch comparisons of the synthesis showed high repeatability, with a mean hydrodynamic radius of 87 nm for the main population of nanoparticles in the medium. Complementary information on the AgNPs characterization can be seen in Figures S2 and S3.

### Variable screening

**Fig. 2:**
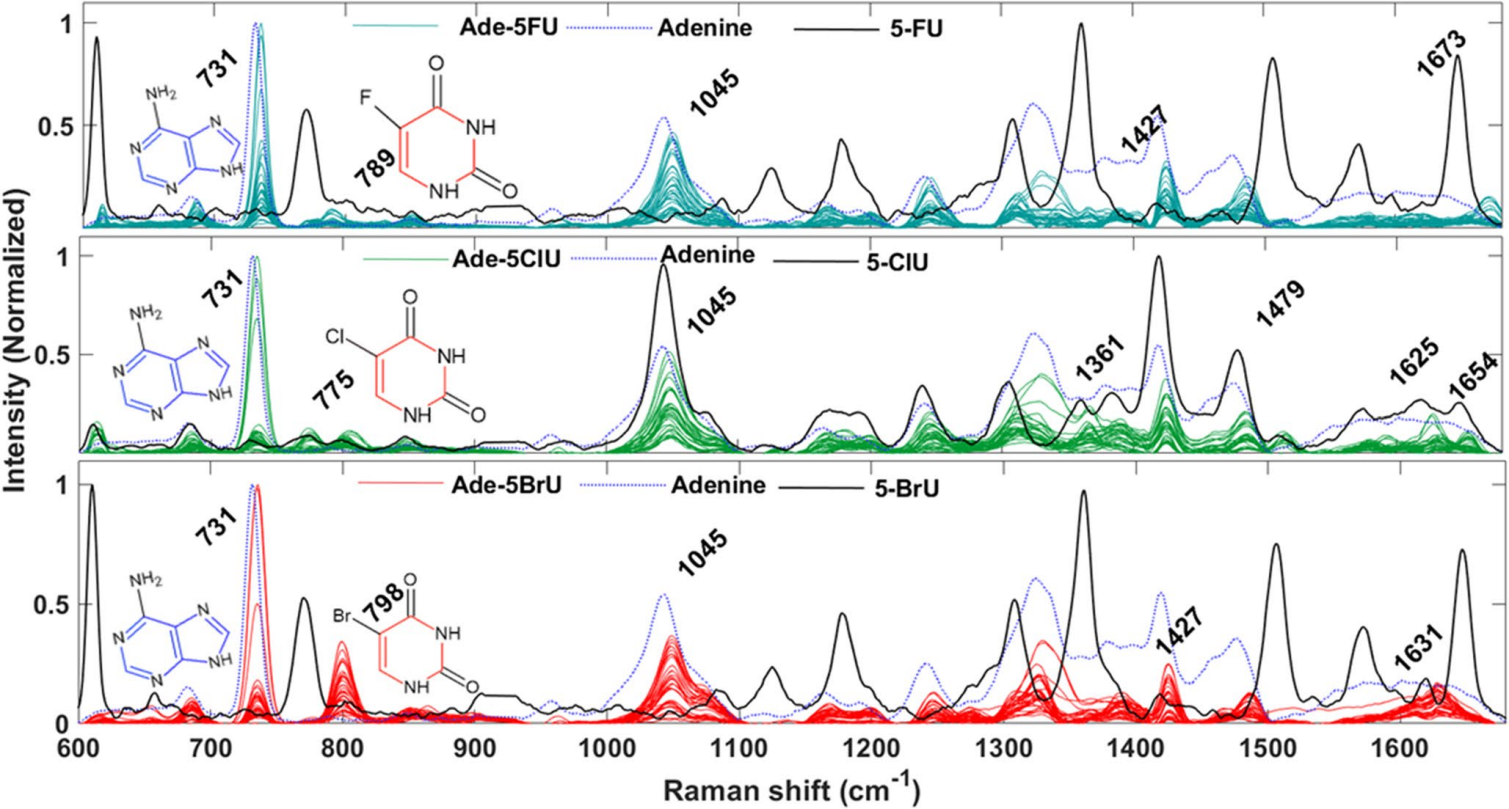
SERS spectra of the three base pairs studied: Ade-5FU, Ade-5ClU, and Ade-5BrU. The SERS spectra of adenine and each halogenated 5 C derivative are included for comparison. The vibrational modes used in the DOE methodologies are illustrated next to each band (731 cm^− 1^ for adenine, and ca. 780 cm^− 1^ for each halogenated derivative). Other band assignment: C-H, bending (958, 1323, and 1360 cm^− 1^), ring stretching (1040 cm^− 1^), NH_2_ rocking (~ 1040 cm^− 1^), C = C stretching (1571 cm^− 1^), and C = O stretching (1647 cm^− 1^)

The SERS spectra obtained from the FFD experiments (values in Table 1) are shown in Fig. 2, with their main band assignments.

By examining Table 1, it was observed that for the Ade-5FU pair, an increase in the volumes of both adenine and NaCl results in higher precision (lower STD values in band intensity). In contrast, for the 5-FU volume, the STD diminished when the spectra were measured at the lowest level. The Ade-5ClU base pair, as indicated by the variables, shows that low volumes of 5-ClU, adenine, AgNPs, and NaCl yield higher precision, as measured by the STD. As expected, the variables for each system will not necessarily be affected in the same way, since each halogen exerts a distinct influence on the electronic and steric structure of the pair, thereby modulating its interaction with the nanoparticle [25].

**Fig. 3:**
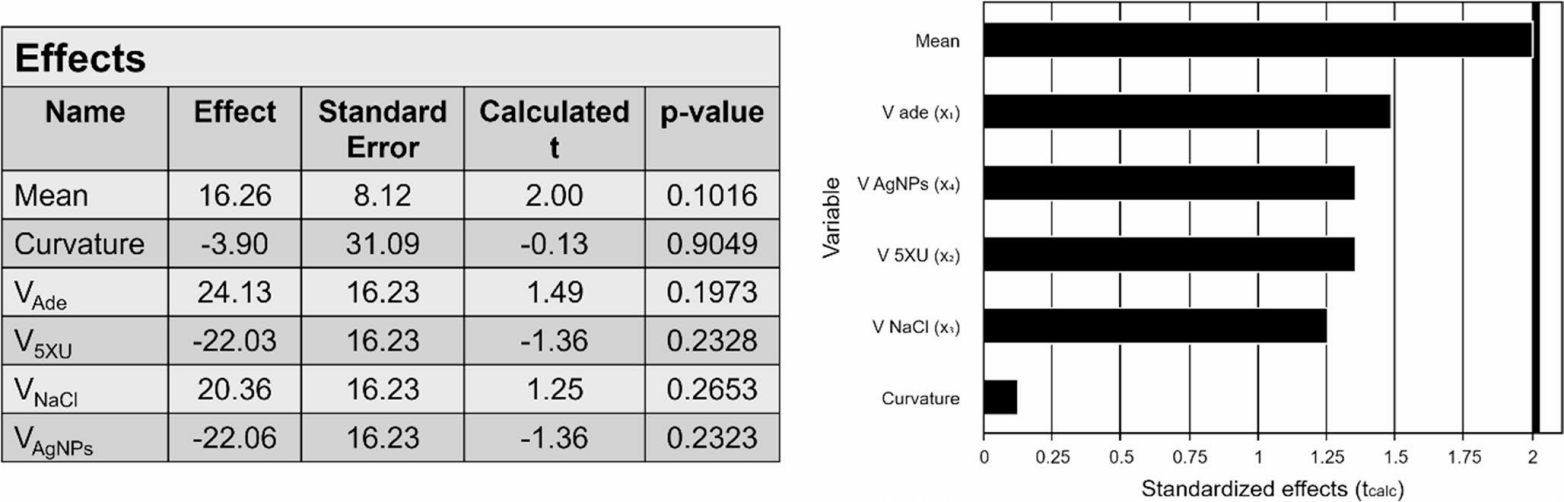
Effects for Ade-5ClU under four experimental conditions (volumes of adenine, 5-XU, NaCl, and AgNPs)

**Fig. 4:**
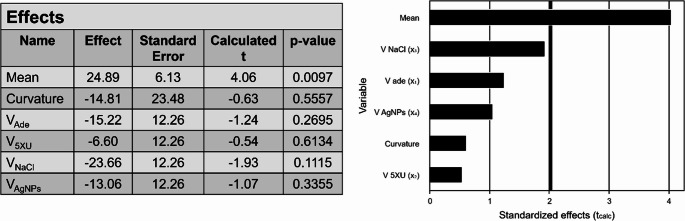
Effects for Ade-5BrU under four experimental conditions (volumes of adenine, 5-XU, NaCl, and AgNPs)

**Fig. 5:**
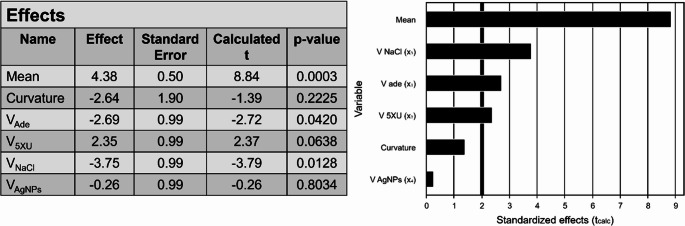
Effects for Ade-5FU under four experimental conditions (volumes of adenine, 5-XU, NaCl, and AgNPs)

As observed in Figs. 3 and 4, for Ade-5ClU (775 cm^− 1^) and Ade-5BrU (798 cm^− 1^), the volumes of adenine, 5-XU, NaCl, and AgNPs were not statistically significant, at 90% confidence (p-value > 0.1). On the other hand, and as shown in Fig. 5, for Ade-5FU (789 cm^− 1^), the significant variables with 90% confidence were the volumes of NaCl (p-value = 0.0128), adenine (p-value = 0.0420), and 5-FU (p-value = 0.0638). Additionally, the volume of AgNPs was not significant (p-value = 0.8034). Based on this, to proceed with the study (optimization stage), the selected variables were the volumes of NaCl and 5-XU. Also, the volume of adenine was fixed at 150 µL for Ade-5-FU and Ade-5BrU, while for Ade-5ClU, this value was 100 µL. It is important to highlight that this difference in the fixed volume of adenine reflects slight differences in how this species governs the orientation of its complementary pair (each 5-XU here) on nanostructured silver, as we have hypothesized.

### Optimization

Table 2 presents the variable levels for each experiment, and the corresponding STD values for the ring-breathing bands obtained for the three base pairs studied (Ade-5FU, Ade-5ClU, and Ade-5BrU). For Ade-5FU, STD ranged from 0.1 (3.43 µL of 5FU and 60 µL of NaCl) to 2.17 (20 µL of 5FU and 20 µL of NaCl). For Ade-5ClU, STD ranged from 0.08 (20 µL of 5ClU and 20 µL of NaCl) to 6.36 (60 µL of 5ClU and 60 µL of NaCl). For Ade-5BrU, STD ranged from 0.29 (200 µL of 5BrU and 100 µL of NaCl) to 0.93 (150 µL of 5BrU and 60 µL of NaCl). The STD values obtained in these experiments were markedly reduced, as detailed in Table 2, suggesting that the variable screening stage allowed significantly minimizing sources of variability, thereby shortening the path to more reproducible SERS spectra for the three 5-halouracil species.

Mathematical models were used to predict the STD of Ade-5FU (Eq. 3), Ade-5ClU (Eq. 4), and Ade-5BrU (Eq. 5) as a function of the coded values of the 5-XU volumes (x_1_) and NaCl volume (x_2_).

3 $$\begin{array}{c}\mathrm{A}\mathrm{d}\mathrm{e}-5\mathrm{F}\mathrm{U} = \mathrm{0,64} - \mathrm{0,16} {\mathrm{x}}_{1} - \mathrm{0,02} {{\mathrm{x}}_{1}}^{2}\\ - \mathrm{0,24} {\mathrm{x}}^{2}+ \mathrm{0,04} {{\mathrm{x}}_{2}}^{2} + \mathrm{0,48} {\mathrm{x}}_{1} {\mathrm{x}}_{2}\end{array}$$

4 $$\begin{array}{c}\mathrm{A}\mathrm{d}\mathrm{e}-5\mathrm{C}\mathrm{l}\mathrm{U} = \mathrm{3,53} + \mathrm{0,31} {\mathrm{x}}_{1} - 1 {{\mathrm{x}}_{1}}^{2}\\ + \mathrm{0,20} {\mathrm{x}}_{2} - \mathrm{1,37} {{\mathrm{x}}_{2}}^{2} + \mathrm{0,54} {\mathrm{x}}_{1} {\mathrm{x}}_{2}\end{array}$$

5 $$\begin{array}{c}\mathrm{A}\mathrm{d}\mathrm{e}-5\mathrm{B}\mathrm{r}\mathrm{U}= {\mathrm{Y}}_{1} = \mathrm{0,69} - \mathrm{0,02} {\mathrm{x}}_{1} - \mathrm{0,10} {{\mathrm{x}}_{1}}^{2}\\ - \mathrm{0,12} {\mathrm{x}}_{2} - \mathrm{0,03} {{\mathrm{x}}_{2}}^{2} + \mathrm{0,01} {\mathrm{x}}_{1} {\mathrm{x}}_{2}\end{array}$$

Although the low values of the coefficient of determination (R^2^) in Table 5 are low, indicating that the models cannot be considered valid, for most CCRD experiments, the STD values fell within a narrow range (0.29–0.93), indicating that within-group variation was much narrower than for Ade-5FU (2.0) and Ade-5ClU (6.3). The above-mentioned higher affinity of Ade-5BrU could account for the low variation in band intensity across the different experimental conditions, since most base-pair species interacting with the hotspot regions would have a lower probability of changing their position or orientation. From an experimental design perspective, the lower variation in response values compared to the other two base pairs suggests that a wider range of experimental conditions could be tested to achieve sharper optimization. This was not conducted in this work, as the values already exhibited low variability in the reproducibility of SERS spectra across the experimental ranges studied. Moreover, these results were already promising for the development of further quantitative approaches. This will be discussed in more detail in the upcoming section.

**Table 5:**
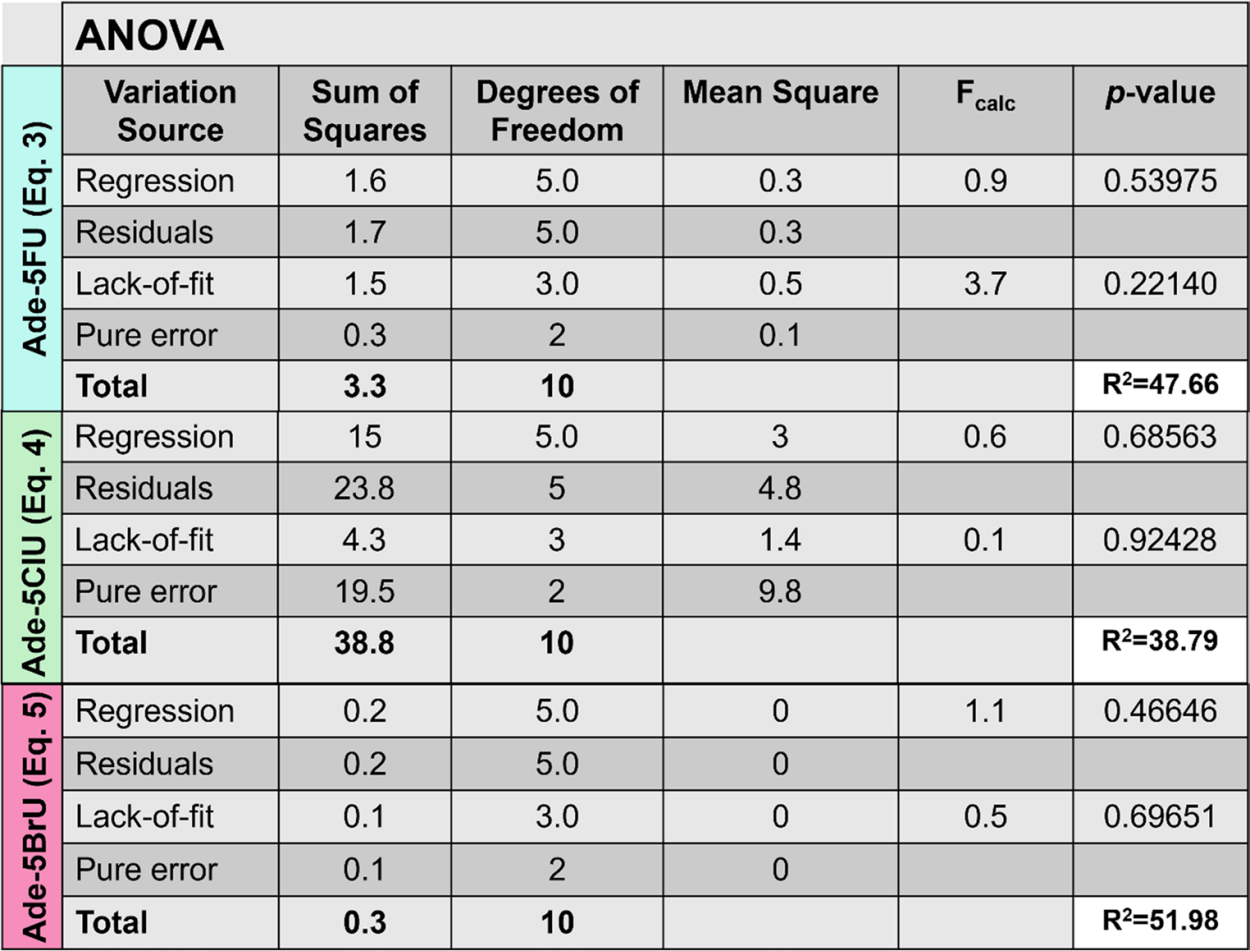
Analysis of variance (ANOVA) of a predictive model for Ade-5FU (Eq. 3), Ade-5ClU (Eq. 4), and Ade-5BrU (Eq. 5)

**Fig. 6:**
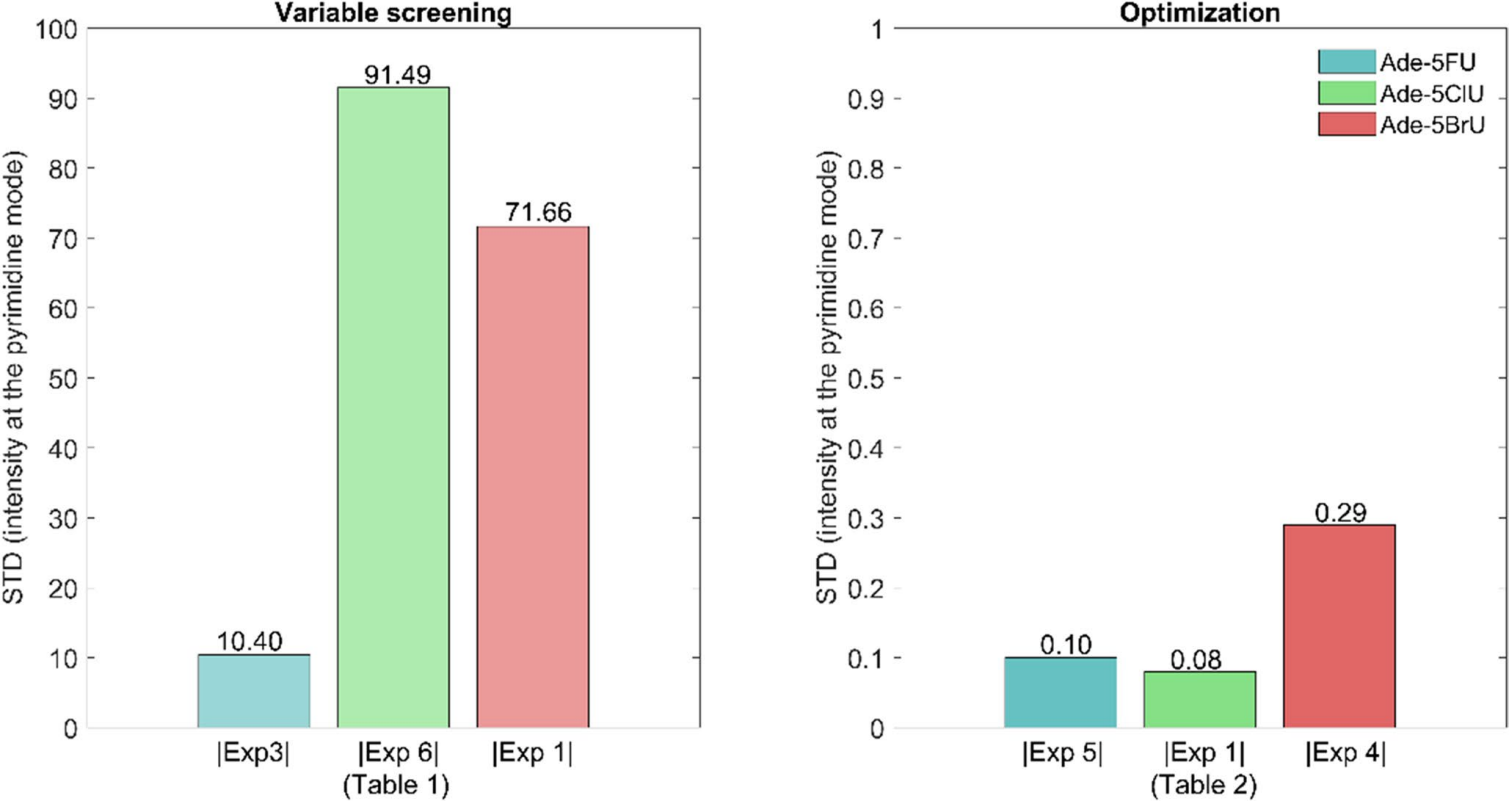
Comparative bar plot of the STD values obtained during the variable screening and optimization stages (*y*-axis scales are different to ease visualization)

To quantitatively assess the extent of the optimization procedure, a comparative bar plot was created, as shown in Fig. 6. Observe that the STD values obtained after optimization were significantly lower than those calculated before the variable screening stage. For the Ade-5FU pair, the STD decreased by almost 100-fold compared with the non-optimized condition (0.10 vs. 10.40). For the Ade-5ClU, this decrease was about 1000-fold. A similar tendency was observed for Ade-5BrU, with a decrease of approximately 300-fold.

### Experimental validation

To conclude the DOE workflow, validation experiments for each base pair were conducted under the optimized experimental conditions depicted in Table 2, and their STD value were calculated. For the three cases, the obtained STD values were found to be at the same order of magnitude as in the optimization stage (0.267, 0.076, and 0.442 for Ade-5FU, Ade-5ClU, and Ade-5BrU, respectively), discussed in Sect.  3.3. Considering the state of the art in quantitative SERS and the scientific community’s efforts to circumvent the low reproducibility, these results are satisfactory and can be further strengthened and refined.

### Multivariate exploratory analysis

**Fig. 7:**
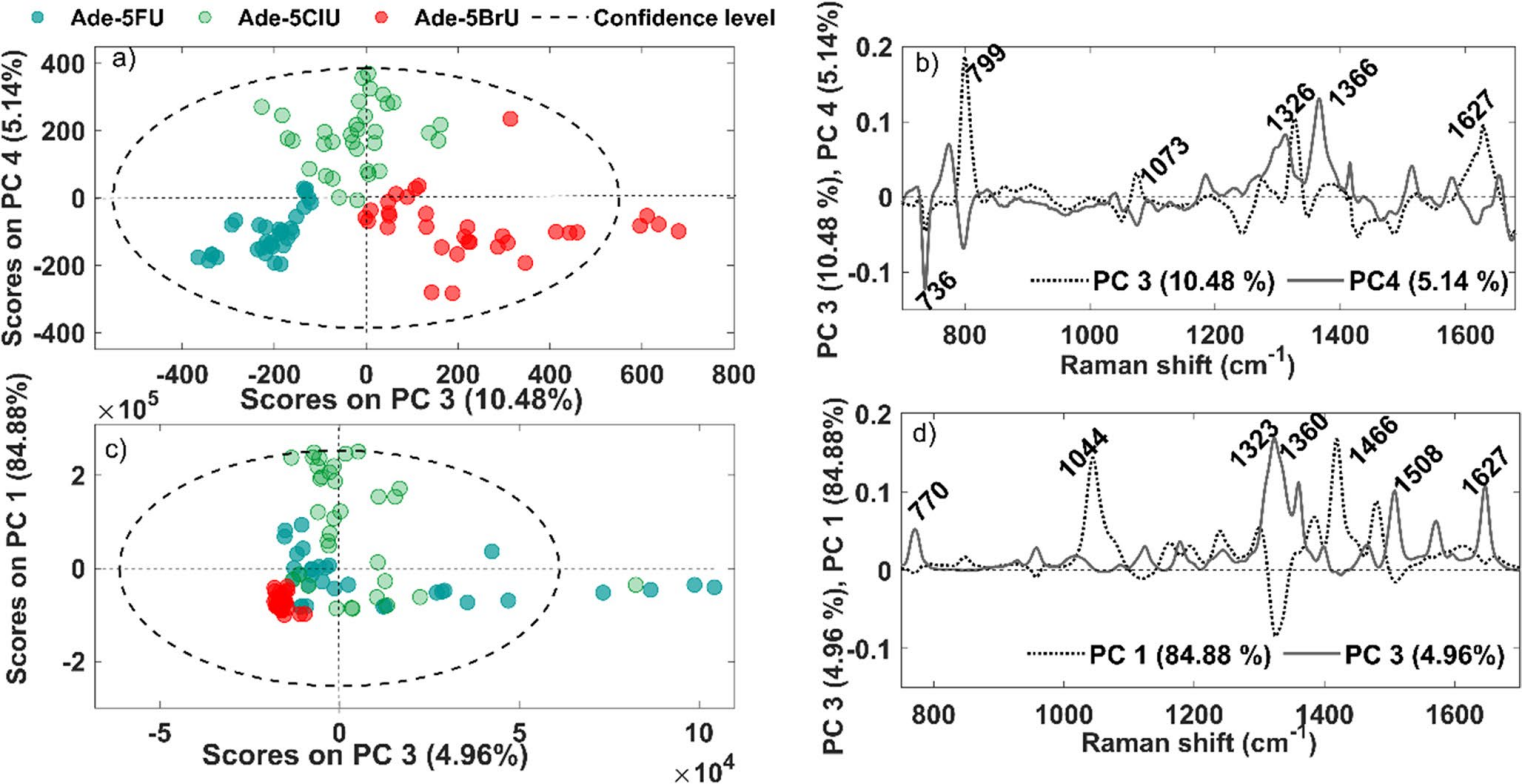
Exploratory analysis through PCA: **a**) and **c**) score plots for the three base pairs (Ade-5XU, Ade-5ClU, and Ade-5BrU; **b**) loading plot of PC3 and PC4 (FFD methodology), and **d**) loading plot of PC1 and PC3 (CCRD methodology)

**Fig. 8:**
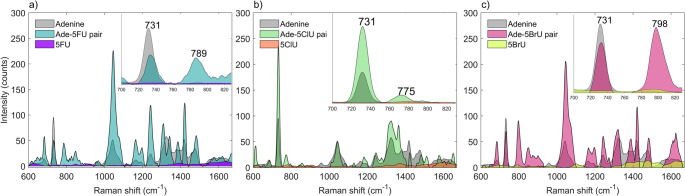
SERS spectra from control experiments, showing the pairs (**a**) Ade-5FU; (**b**) Ade-5ClU; (**c**) Ade-5BrU. The adenine and 5-XU concentrations used in these measurements were 5 × 10^− 5^ and 1 × 10^− 4^ mol L^− 1^, respectively. The inset plots show the spectral range between 700 and 800 cm^− 1^, highlighting the ring-stretching mode for each species pair

A complementary exploratory PCA analysis was performed to provide a final overview of the behavior of the three base pairs and to verify whether the optimized experimental conditions will allow their discrimination based on their SERS spectra. In addition, a multivariate approach allows us to determine whether the conclusions drawn from analyzing a single band (in the DOE methodologies) are representative of the entire spectrum. From the score plot obtained with the FFD methodology, shown in Fig. 7a, it is already clear that there are significant differences, indicating a trend of the 5-halouracils along PC3 and PC4. It is worth noting that the Ade-5BrU pair is the only species grouped at the positive side of PC3. The loading plot of this PC (Fig. 7b) shows that the greater influence is due to the 5-BrU modes (ring breathing, -C-H, and -C = O), which is consistent with the discussion in the later subsection. The 5-FU trend is driven primarily by the main adenine mode (731 cm^− 1^) and, secondarily, by its own ring-breathing mode at 789 cm^− 1^. This makes complete sense given the apparently strong influence of adenine, especially on this halouracil as an additional SERS enhancer (See the spectra with and without adenine shown in Fig. 8). On the other hand, the Ade-5ClU pair, located at the positive region of PC4, shows a stronger influence of the -C-H bending modes of the 5-XU, and secondly, of the ring breathing (774 and 799 cm^− 1^) modes.

After the variable screening stage, the CCRD-PCA scores plot (Fig. 7c) showed a more depurated behavior, as the Ade-5BrU pair is highly concentrated in the negative region of PC3, which, in this case, is strongly influenced by the C-H bending vibrational modes at 1323 and 1360 cm^− 1^. Also, the Ade-5FU shows higher values on PC3, driven by modes at 1044 and 1466 cm^− 1^, which correspond to the NH_2_ and N-H moieties. Finally, the Ade-5ClU pair is clearly moving towards positive PC1 values, governed by the 1323, 1647, and 770 cm^− 1^ bands, assigned to -C-H bending, -C = O, and ring-breathing vibrational modes. This change in the behavior from the variable screening to the optimization stage is quite interesting because distinctive bands along the entire spectra could be identified for each base pair, which means, in the first place, that our initial univariate approach (intensity STD at a single band) is, indeed, representative of the entire spectrum; and, secondly, that the multivariate exploratory analysis let to extract distinctive bands associated to the behavior of each base pair, thus allow their identification almost as a fingerprint. This last statement is closely related to the well-known and expected changes in polarizability that depend on the nature of the C5 substituent. From a structural point of view, it is interesting to note that, upon optimization, the -NH_2_ rocking band at 1044 cm^− 1^ became highly influential (PC1 loadings in Fig. 7d), whereas it was weak during the variable screening procedure. This fact, from the perspective of SERS, indicates an increased importance of hydrogen-bond interactions that seem to stabilize the base pair, as discussed in the section ahead [25, 36]. 

It is important to remember that the original spectra of the three species are highly similar, as shown in Figs. 1 and 2SI, and that it is quite difficult to identify each species solely by visual inspection of its spectra.

### Co-enhancement effect and control experiments

In the systems studied in this work, adenine acts as an additional signal enhancer, apparently dependent on hydrogen bonds between the NH2 and NH moieties of adenine and the 5-XU, thereby inducing a more favorable orientation of the uracil derivatives. Rueda et al. have previously suggested that this arises from a spatial rearrangement of the molecules on the metal surface to facilitate these interactions, leading to cooperative co-adsorption [38]. Zhang et al. studied the co-adsorption of adenine with an uracil analog (thymine) and associated the bands’ intensities with different ring-tilt angles in both species [22]. Their results matched experimental observations reported in similar works [23, 39, 40], and also the behavior observed for the three base pairs studied here. To illustrate this, control experiments were run to show that the 5-XU spectra in the absence of adenine have a very low signal-to-noise ratio, whereas paired with adenine, the signal is greatly intensified, as demonstrated in Fig. 8. This is even more evident in the region between 700 and 800 cm^− 1^, shown in the inset plots, where the ring-stretching modes of purine and pyrimidine appear as their characteristic bands.

## Conclusions

From an analytical point of view, using DOE methodologies was a very suitable choice to minimize the effects of the main parameters influencing the SERS response. This is relevant to the SERS analytical research area because achieving high reproducibility in SERS spectra remains one of the most difficult bottlenecks to overcome in quantitative applications, as repeatedly discussed in the literature. The results herein demonstrated that a variable screening methodology followed by optimization, carrying out only 22 experiments overall, is a shorter path to reach the best experimental conditions to bring out improved signals, i.e., spectra with high signal-to-noise ratio, and, more importantly, circumvent inherent low reproducibility in quantitative SERS applications using colloidal nanoparticles. For the three 5-XU species, the STD decreased by at least 100-fold and up to 1000-fold at the highest. The validation experiments achieved STD values comparable to those obtained during the optimization, and our approach has proven promising for application to analogous systems focused on analytical SERS. Besides, the approach used in this work significantly reduces time and reagent consumption, a critical concern when establishing experimental conditions for developing an analytical methodology. The compounds studied, belonging to a series of species that differ by only one atom, exhibited different behavior under the experimental conditions evaluated, yet they could still be optimized simultaneously. Finally, it is important to emphasize that the strategy presented here could be further improved to achieve even lower STD values.

## Supplementary Information

Below is the link to the electronic supplementary material.

Supplementary Material 1

## Data Availability

Data will be available upon reasonable request.
