# Supplementary material for: A shorter path to circumventing the low reproducibility of SERS spectra through variable screening and optimization methodologies
Source: Mikrochim Acta. 2026 Jul 28;193(8):579. doi: 10.1007/s00604-026-08306-x (PMC13415297; doi:10.1007/s00604-026-08306-x)
Supplement: Supplementary file 1 — Supplementary Material 1 [file 604_2026_8306_MOESM1_ESM.docx]

**A SHORTER PATH TO CIRCUMVENTING THE LOW REPRODUCIBILITY OF SERS SPECTRA BY VARIABLE SCREENING AND OPTIMIZATION METHODOLOGIES**

Supporting Information


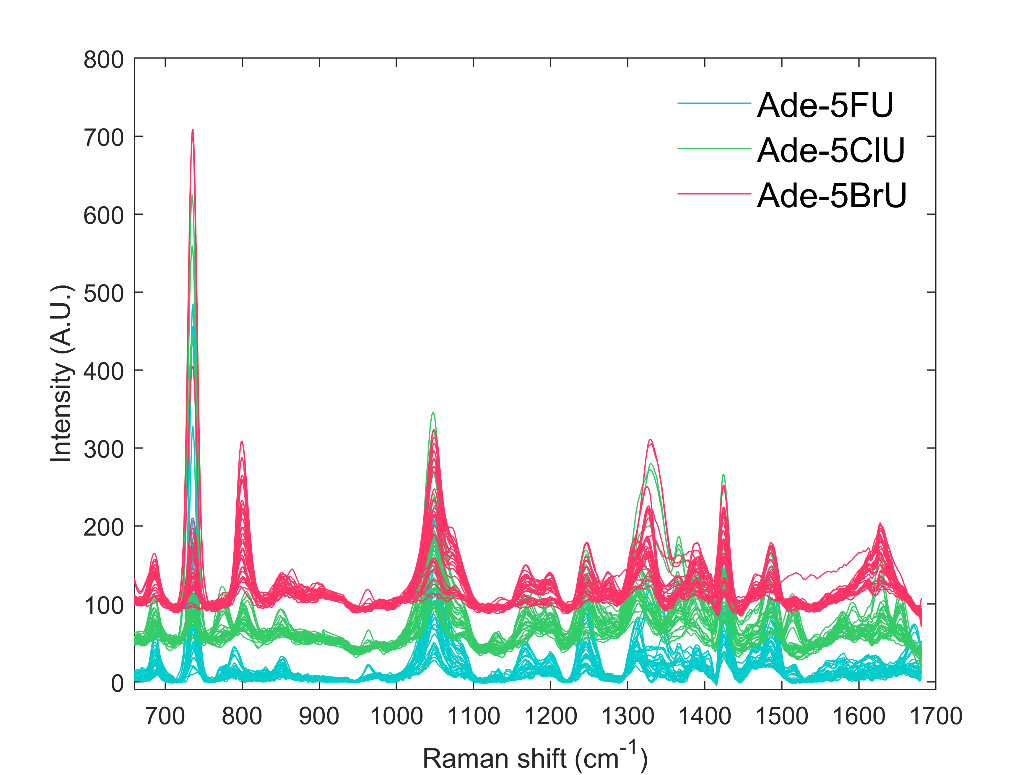


Figure S1. SERS spectra for the Ade-5XU base pairs on AgNPs.


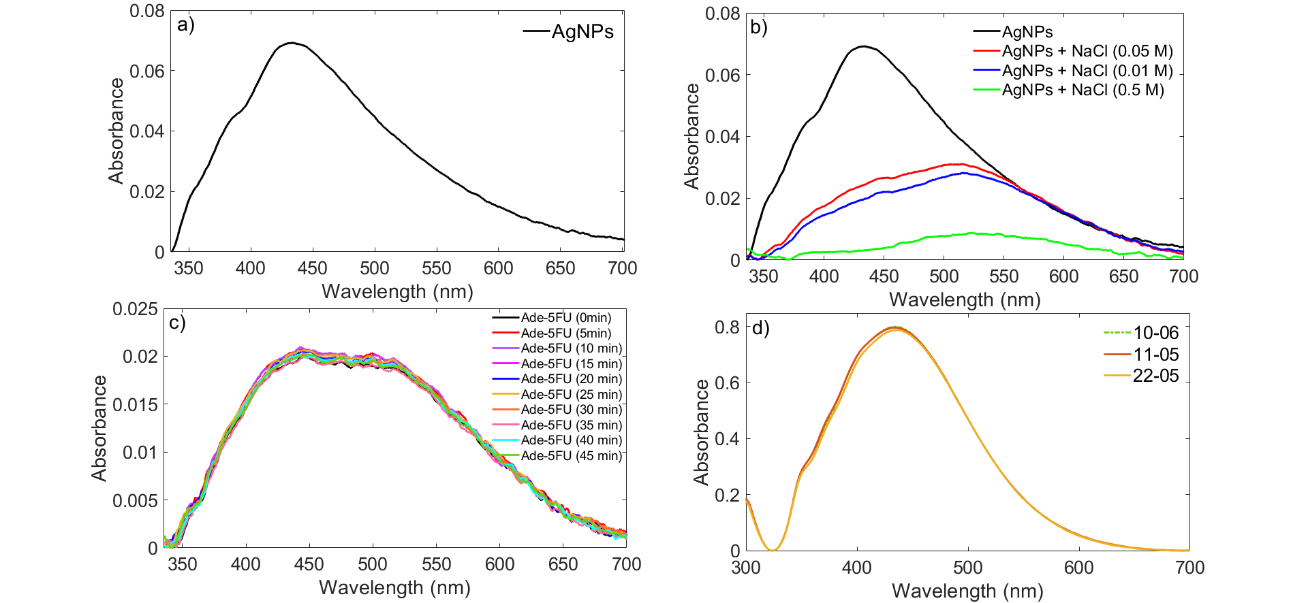


Figure S2. Extinction spectra of a. AgNPs; b. AgNPs under different ionic strength; c. AgNPs activated with NaCl and mixed with the base pair Ade-5FU for 45 min; c. AgNPs from three different batches.


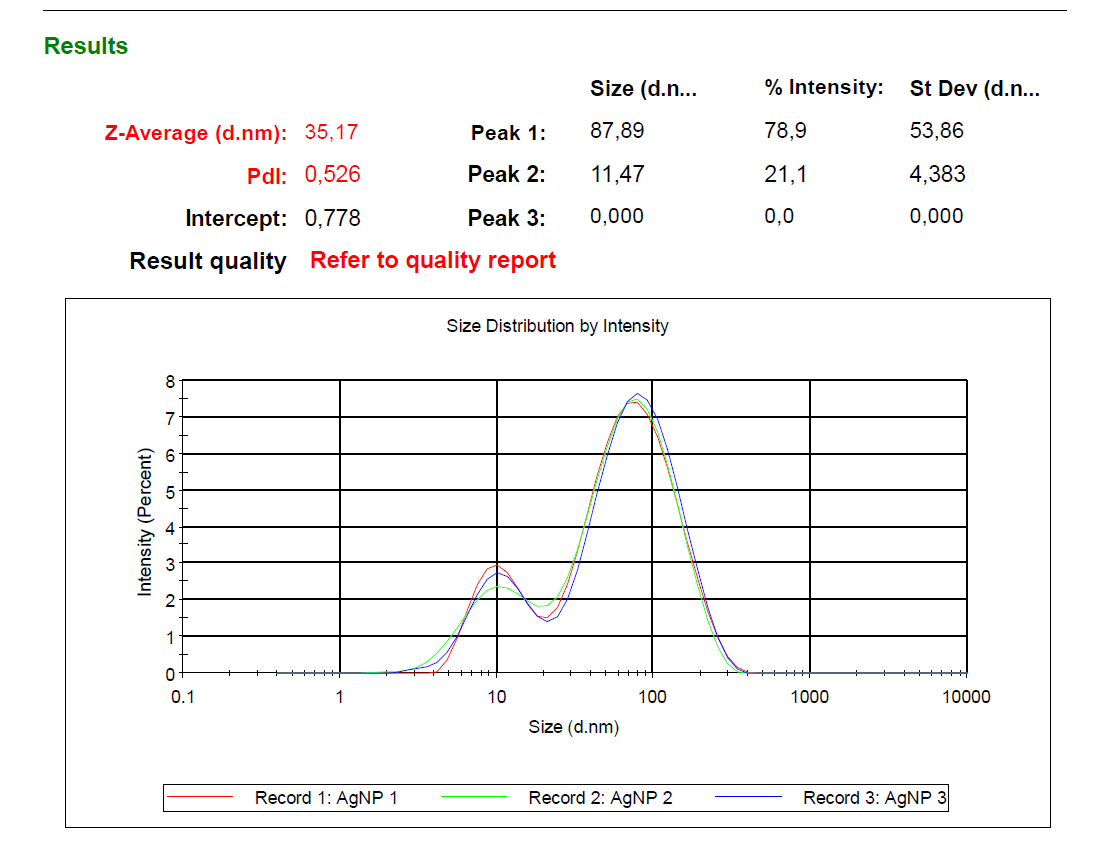


Figure S3. Results of the DLS analysis described in Section 2.3.

Table 1SI. Standard deviation values for the bands at and 789, 775, and 798 cm-1. The FFD included four variables.

| **Experiments** | **Standard deviation of the intensity at the pyrimidine mode of each pair** | | |
| --- | --- | --- | --- |
|  | **Ade-5FU(789)** | **Ade-5ClU(775)** | **Ade-5BrU(798)** |
| 1(-1,-1,-1,-1) | 6.40 | 1.75 | 71.66 |
| 2 | 2.49 | 12.45 | 16.02 |
| 3 | 10.40 | 2.91 | 41.62 |
| 4 | 5.72 | 7.20 | 17.57 |
| 5 | 2.05 | 3.39 | 2.67 |
| 6 | 1.87 | 91.49 | 22.40 |
| 7 | 4.04 | 8.70 | 14.04 |
| 8 | 2.05 | 2.15 | 13.12 |
| 9 | 2.57 | 16.32 | 18.05 |
| 10 | 3.67 | 12.75 | 8.31 |
| 11 | 2.93 | 13.83 | 26.08 |

Table 2SI-Standard deviation values for the bands at and 789, 775, and 798 cm-1, obtained from the CCRD methodology, and related to the heatmap in Figure 4.

| **Experiments** | **Standard deviation of intensity at (772 and 771) cm-1** | | |
| --- | --- | --- | --- |
|  | **Base Pairs** | | |
|  | **Ade-5FU** | **Ade-5ClU** | **Ade-5BrU** |
| 1 | 2.17 | 0.08 | 0.79 |
| 2 | 0.15 | 0.26 | 0.69 |
| 3 | 0.61 | 0.68 | 0.34 |
| 4 | 0.50 | 3.02 | 0.29 |
| 5 | 0.10 | 1.69 | 0.51 |
| 6 | 0.72 | 1.66 | 0.52 |
| 7 | 0.77 | 1.56 | 0.69 |
| 8 | 0.27 | 0.33 | 0.60 |
| 9 | 0.62 | 6.36 | 0.59 |
| 10 | 1.01 | 4.05 | 0.54 |
| 11 | 0.28 | 0.18 | 0.93 |
